# Heterogeneous treatment response in metastatic breast cancer: discordance between FDG-PET and CT classification

**DOI:** 10.1186/s40644-026-01119-4

**Published:** 2026-08-26

**Authors:** Vilma Landfors Andersson, Kristin Johnson, Ida Skarping

**Affiliations:** 1 Faculty of Medicine, Lund University, Lund, Sweden; 2 Department of Translational Medicine, Diagnostic Radiology, Lund University, Malmö, Sweden; 3 Department of Imaging, Physiology, and Nuclear Medicine, Skåne University Hospital, Region Skåne, Sweden; 4 Division of Oncology, Department of Clinical Sciences Lund, Lund University, Lund, Sweden; 5 Centre for Interdisciplinary Research on Cancer and Equity in Women, Lund University, Lund, Sweden

**Keywords:** Metastatic breast cancer, Heterogeneous response, Treatment evaluation, FDG-PET, CT

## Abstract

**Background:**

Heterogeneous treatment response, characterized by simultaneous regression of some lesions and progression of others within the same patient, is increasingly recognized in metastatic breast cancer (mBC) and may reflect biological heterogeneity. However, this response pattern is not captured by current response assessment frameworks. We aimed to determine the prevalence of heterogeneous treatment response on FDG-PET and mixed response on CT during mBC systemic therapy.

**Methods:**

This retrospective study included 51 female patients with mBC, treated between November 2017 and November 2025, each undergoing ≥ 2 FDG-PET/CT scans. Response was assessed according to RECIST 1.1 (CT) and PERCIST (FDG-PET), with using study‑specific definitions for heterogeneous/mixed response informed by literature.

**Results:**

Heterogeneous metabolic response was observed in 30% of all follow‑up FDG‑PET examinations (40/132). These examinations accounted for 60% (40/67) of the scans classified as progressive metabolic disease (PMD) according to PERCIST. Among CT-examinations, 65 of 141 (46%) follow-up examinations had measurable disease; none of these scans (0/65, 0%) fulfilled the mixed response criteria.

**Conclusion:**

In this real-world cohort of mBC undergoing systemic therapy, FDG-PET identified heterogeneous treatment response in a substantial proportion of patients; however, under current PERCIST criteria, these cases were uniformly classified as PMD. In contrast, using RECIST-inspired criteria, CT did not classify any patient as mixed response. These findings suggest that existing frameworks may not fully capture intra-patient response heterogeneity. As heterogeneous response is not an established PERCIST response category, further work is needed to determine how such patterns should be defined and incorporated into future response assessment frameworks.

**Clinical trial number:**

Not applicable.

**Graphical Abstract:**

**Figure:**
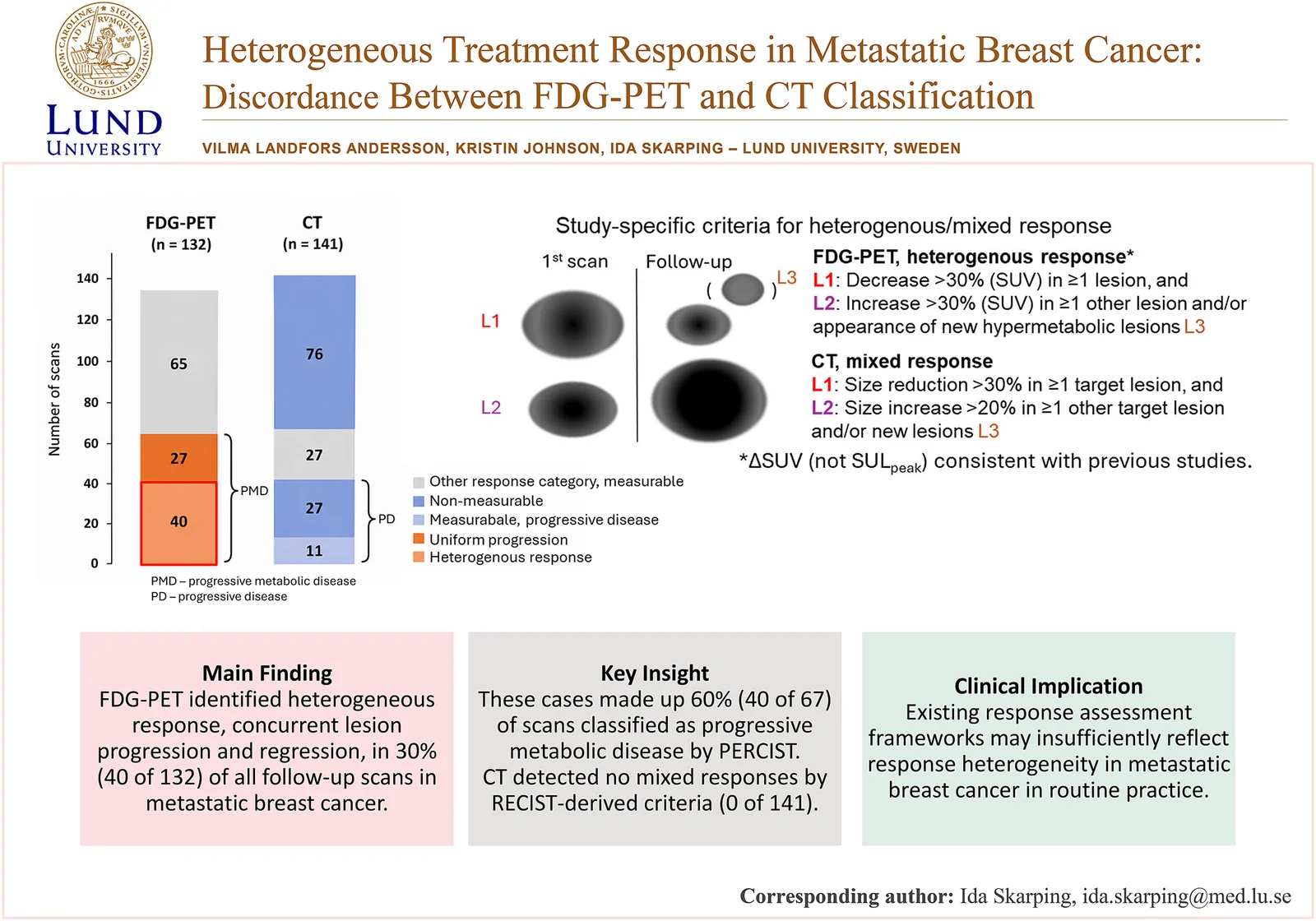

**Supplementary information:**

The online version contains supplementary material available at 10.1186/s40644-026-01119-4.

## Key points

− Response heterogeneity during systemic therapy in metastatic breast cancer may not be captured by current response criteria.

− In real-world data, heterogeneous metabolic response comprised 60% of PERCIST-defined progression, whereas RECIST-derived criteria detected no mixed responses on CT.

− RECIST 1.1 and PERCIST frameworks may insufficiently capture intra-patient heterogeneity, potentially limiting treatment assessment in metastatic breast cancer.

## Introduction

Although most patients with breast cancer are diagnosed at an early stage and treated with curative intent, approximately 20–30% ultimately develop metastatic disease [1]. Owing to advances in systemic therapies, including targeted and biologically tailored treatments, a subset of patients with distant metastatic breast cancer (mBC) now experience prolonged survival with multiple lines of therapy. As a result, disease trajectories have become more variable, ranging from indolent to rapidly progressive patterns. Accurate response assessment is central to clinical decision-making within this evolving and increasingly complex therapeutic landscape, often characterized by sequential and parallel lines of treatment. Imaging therefore plays a pivotal role, providing longitudinal evaluation of treatment effects that informs both clinical trial endpoints and routine clinical practice.

In mBC, for systemic treatment response assessment, guidelines provide limited recommendations regarding preferred imaging modality [2]. Commonly used techniques include computed tomography (CT) and ^18^F fluorodeoxyglucose positron emission tomography (FDG-PET) [2]. Each modality has inherent limitations and partially overlapping diagnostic capabilities. CT response evaluation typically follows Response Evaluation Criteria in Solid Tumours (RECIST) 1.1, which classifies response into four categories—complete response (CR), partial response (PR), stable disease (SD), and progressive disease (PD) [3]—while FDG-PET assessments commonly use PET Response Criteria in Solid Tumours (PERCIST), which likewise defines four metabolic response categories: complete metabolic response (CMR), partial metabolic response (PMR), stable metabolic disease (SMD), and progressive metabolic disease (PMD) [4–6].

Patterns of metastatic involvement also influence modality performance. Bone represents the most frequent site of distant metastatic spread in breast cancer, affecting up to 70% of patients with advanced disease, followed by metastases to the lung, liver, and brain [7]. Patterns of dissemination vary by tumor subtype and are differentially visualized across imaging techniques [8, 9]. Lesion burden may additionally differ substantially between modalities, an issue particularly relevant in oligometastatic disease [10] and potentially in the evaluation of lesion-level treatment response. For example, lesion counts on CT are often lower than those detected with FDG-PET [11]. CT may also be limited in accurately characterizing bone and liver metastases [12, 13], while invasive lobular carcinoma can be challenging to detect with both CT and FDG-PET [8, 14].

mBC is characterized by spatial and temporal heterogeneity, with lesions within the same patient often demonstrating divergent treatment responses. In FDG-PET, this phenomenon is frequently referred to as *heterogeneous* or *dissociated* metabolic response, describing scenarios in which some lesions respond while others progress [15, 16]. On CT, an analogous concept, commonly termed *mixed response*, is recognized clinically but is not formally incorporated into the RECIST 1.1 framework [15, 16]. Under both RECIST 1.1 and PERCIST, such patients are typically classified as progressive disease, as the appearance of a single new lesion mandates classification as PD or PMD, respectively, regardless of response in other lesions.

Prior studies suggest that heterogeneous metabolic response may be associated with more favorable outcomes than uniform progressive metabolic disease, including prolonged time to clinical progression in both immunotherapy-treated cohorts and bone-predominant mBC [17–19]. These findings likely reflect underlying interlesional genomic heterogeneity and clonal evolution heterogeneity [20]. However, the extent to which current imaging-based response frameworks adequately capture this phenomenon in routine clinical practice remains uncertain.

In this real‑world cohort of mBC, we aimed to analyse FDG‑PET and CT separately to evaluate their ability to capture heterogeneous metabolic and mixed anatomical response patterns, respectively, during systemic therapy. As a secondary objective, paired-scan analyses were performed to compare response classifications between modalities. In addition, we sought to characterize the subgroup exhibiting heterogeneous response by examining key biological and disease features, including histopathological subtype, receptor-derived tumor phenotype, metastatic distribution, and overall lesion burden.

## Methods and materials

This retrospective study included patients with metastatic breast cancer treated at Skåne University Hospital (Sweden) between November 2017 and November 2025 who underwent clinically indicated FDG-PET/CT. In accordance with national practice recommendations [21], FDG-PET examinations were typically reserved for patients with inconclusive or discordant findings on conventional imaging. Eligibility required ≥ 2 FDG-PET/CT examinations. Clinicopathological data were retrieved from electronic medical records. The study complied with the Declaration of Helsinki and was approved by either the Ethics Committees at Lund University or by the Swedish Ethical Review Authority (EPN 2016/417, 2018/753, 2021–05734-02). All participants provided written informed consent.

### Study specific cohort

The primary analyses were performed separately for each modality and included all available follow-up FDG-PET and CT examinations. Time-matched paired follow-up examinations constituted a secondary cohort used exclusively for intermodality comparison.

### Image acquisition

All FDG-PET scans were performed on GE Discovery MI, Discovery 690, or Omni Legend systems (GE Healthcare, USA). Patients fasted ≥ 4 hours before receiving 3.5–4 MBq/kg ^18^F-FDG intravenously, followed by a 60 minute uptake period. PET was acquired from orbitomeatal line to upper thighs. The PET systems were integrated with a 64-slice CT. Intravenous and oral iodine contrast was used. CT examinations not performed in conjunction with PET were carried out on different machines at different hospitals within the Skåne health care region in southern Sweden. These CT examinations were performed as CT thorax and abdomen with intravenous iodine contrast. Oral contrast was not routinely used.

### Heterogenous response criteria

As no standardized criteria for heterogeneous response are currently available, we derived working definitions informed by relevant literature and by considerations of clinical applicability and feasibility. These definitions were applied consistently for all heterogeneous/mixed response assessments in relation to the preceding scan.

- FDG-PET, heterogenous response:

Presence of both:

1. Metabolic decrease >30% (SUV) in ≥1 lesion, and
2. Metabolic increase >30% in ≥1 other lesion and/or appearance of new hypermetabolic lesions [16, 18]

- CT, mixed anatomical response inspired by RECIST 1.1 criteria:

Presence of both:

1. Size reduction >30% in ≥1 target lesion, and
2. Size increase >20% in ≥1 other target lesion and/or new lesions [16]

### Image assessment

During the retrospective, study-specific review of original clinical reports and images, all examinations were assessed in Sectra Picture Archiving and Communication System and in HERMES Hybrid Viewer, with HERMES used specifically for PET interpretation and standardized uptake value (SUV)/standardized uptake value corrected for lean body mass (SUL) measurements. For each patient, the first PET and CT examination within the study window was defined as baseline, regardless of the systemic treatment being administered at that time. Subsequent examinations were evaluated according to RECIST 1.1 (CT), the mixed anatomical response criteria, one-lesion PERCIST (FDG-PET), and the heterogenous response criteria using ΔSUV [3–6, 22]. Specifically, each follow-up examination was formally categorized according to RECIST 1.1 (CT) and PERCIST (FDG-PET), using baseline or nadir as reference in accordance with the respective response frameworks. Subsequently, the study-specific mixed response and heterogeneous response assessments were applied to examinations classified as progressive disease (CT) and progressive metabolic disease (FDG-PET), respectively, based on lesion-level comparison with the immediately preceding examination. All follow-up examinations were categorized as containing measurable or non-measurable disease according to the respective response assessment framework, as this determined eligibility for the heterogeneous/mixed response analyses. Oligometastatic disease was defined as the presence of up to five metastatic lesions (≤5 cm each) involving no more than three organs, regardless of RECIST 1.1 or PERCIST lesion classification [10, 23, 24]. Response assessments were performed by a last semester medical student under supervision, with full access to all imaging data and original clinical reports. All PET categorizations and a random 50% subset of CT assessments were independently checked by two experienced readers (IS and KJ) according to a predefined data monitoring plan.

### Statistics

Continuous variables are summarized as median (range) and categorical as percentages. Group comparisons used Fisher’s exact test for 2 × 2 and sparse contingency tables, and χ^2^ tests for larger tables; a two‑sided α = 0.05 was applied. Data processing and statistical analyses were conducted in R (v4.2.1) and SPSS v28. R code is available on request.

## Results

**Fig. 1:**
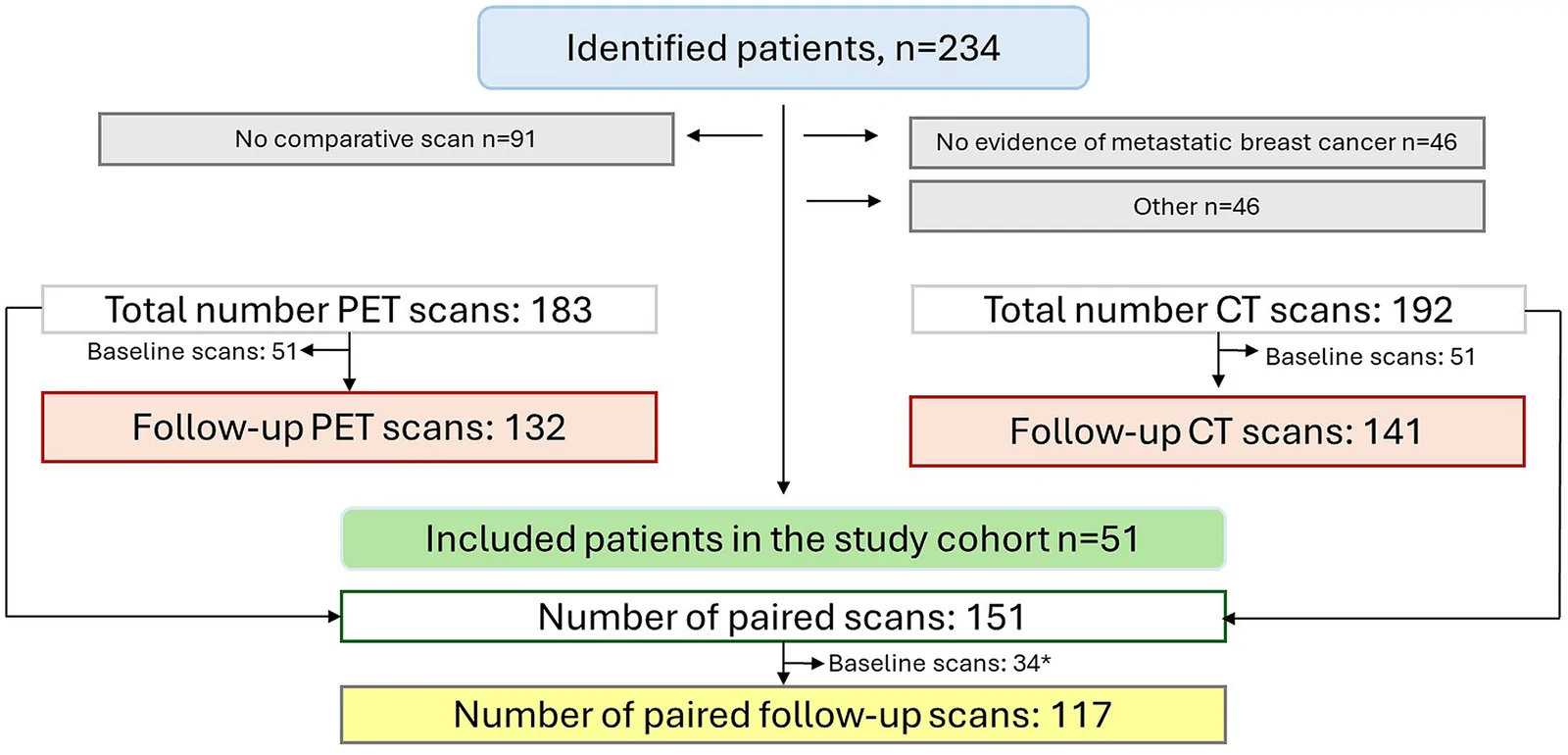
Flowchart of the study cohort (green box), showing follow-up FDG-PET and CT examinations (red boxes) for heterogeneous and mixed response assessment, and time-paired examinations (yellow box) for comparison between modalities. *only 34 paired scans were excluded, as baseline examinations had already been removed due to missing time-matched PET/CT pairs

The 51 patients included in the study contributed a total of 132 follow-up FDG-PET examinations for assessment of heterogeneous metabolic response and 141 follow-up CT examinations for assessment of mixed anatomical response (Fig. 1, red boxes). A subcohort of 117 time-matched paired follow-up FDG-PET and CT examinations was available for intermodality comparison (Fig. 1, yellow box).

### Patient and tumour characteristics

The clinical characteristics of the 51 female patients (median age, 52 years, range 28–83) and the 151 paired FDG-PET and CT scans (Fig. 1) included in this study are summarized in Table 1. Most patients (39/51, 76%) had luminal subtype disease, while five (10%) were HER2-positive and seven (14%) had triple-negative breast cancer. Histopathologically, invasive carcinoma of no special type was most common (40/51, 80%), followed by invasive lobular carcinoma (9/51, 18%).

**Table 1:** Patient. tumour, and scan characteristics

Variable	*N* = 51 study participants
Age at diagnosis of primary BC/metastatic BC (years), median (range)	52 (28–83)/59 (34–83)
Time from primary BC to metastatic BC (years), median (range)	4.0 (0–26)
Type of metastatic BC, N (%)	
-Recurrent	36 (70.6%)
-De novo	15 (29.4%)
Biological subtype primary BC, N (%)	
-Luminal A/B	39 (76.5%)
-HER2-positive	5 (9.8%)
-Triple negative	7 (13.7%)
Histopathological subtype primary BC, N (%)	
-No specific type	40 (80.0%)
-Lobular	9 (18.0%)
-Other	1 (2.0%)
-Missing	1

*Note: Paired‑scan totals differ from response‑category totals due to inclusion of baseline scans

Abbreviations: BC, breast cancer; ER, estrogen receptor; HER2, human epidermal growth factor 2 receptor. Note: Percentages may not sum to 100% due to rounding

### Response heterogeneity

**Fig. 2:**
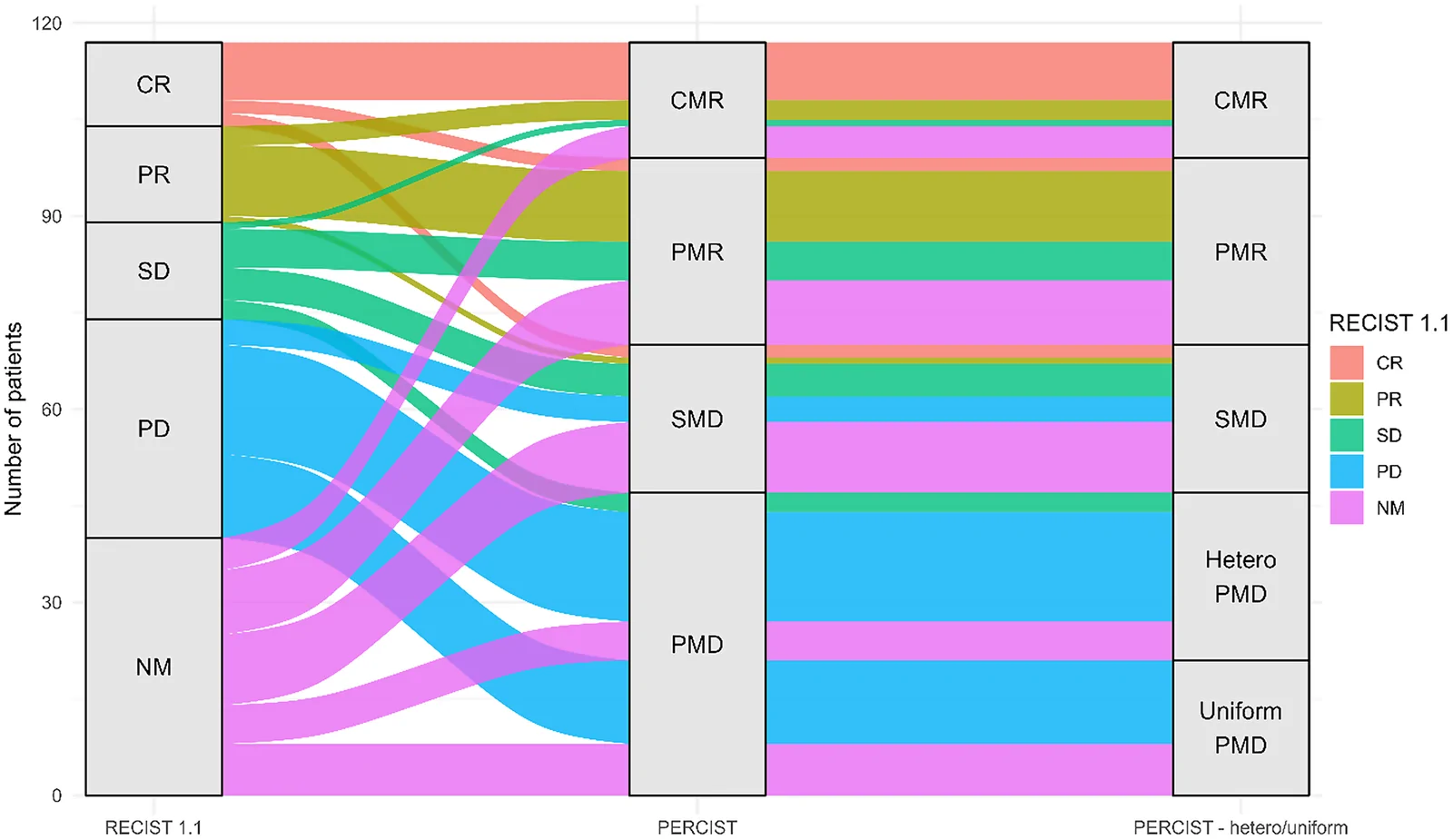
Sankey diagram illustrating the relationship between RECIST 1.1 response categories (left), PERCIST categories (middle), and the modified PERCIST classification (right) for 117 paired follow-up scans (yellow box cohort in Fig. 1). In the modified PERCIST classification, progressive metabolic disease is subdivided into heterogeneous metabolic response and uniform progressive metabolic disease. No patients classified as progressive disease on CT showed RECIST 1.1-derived mixed response. Abbreviations: CR, complete response; PR, partial response; SD, stable disease; PD, progressive disease; NM, non-measurable disease; CMR, complete metabolic response; PMR, partial metabolic response; SMD, stable metabolic disease; PMD, progressive metabolic disease

A total of 132 follow‑up FDG‑PET examinations were available for analysis (Fig. 1, red box). Heterogeneous metabolic response was observed in 30% (40/132) of follow‑up scans, all of which were classified as PMD according to PERCIST. These accounted for 60% (40/67) of all scans categorized as PMD (Table 2). Among the 117 paired follow‑up examinations (Fig. 1, yellow box), the 26 scans categorized as exhibiting heterogeneous metabolic response were predominantly classified as progressive disease on corresponding CT/RECIST 1.1 assessment (17 scans), while six were classified as non‑measurable and three as stable disease (Fig. 2). There was a trend toward a higher frequency of luminal mBC in the heterogeneous metabolic response subgroup (32/40, 80%) compared to the uniform progressive disease subgroup (18/27, 67%) (*p* = 0.08) (Table 3). The metastatic site distribution spanned multiple locations and was broadly comparable between scans demonstrating heterogeneous metabolic response and those showing uniform progressive metabolic disease (Table 3; a detailed description is provided in Additional File 1).

**Table 2:** Distribution of heterogeneous (FDG-PET) and mixed (CT) responses versus other PERCIST and RECIST 1.1 response categories, with emphasis on progressive (metabolic) disease

		Heterogenous response	Other response category	Non-measurable disease
FDG-PET	All scans, *N* = 132	40 (30.3%)	92 (69.7%)	0
	** -PMD, 67 scans**	**40 (59.7%)**	27 (40.3%)*	**0**
		Mixed response	Other response category	Non-measurable disease
CT	All scans, *N* = 141	0	65 (46.1%)	76 (53.9%)
	** -PD, 38 scans**	**0**	11 (28.9%)*	**27 (71.1%)**

*Uniform progression

Abbreviations: PMD, progressive metabolic disease; PD, progressive disease

**Table 3:** Clinical and FDG-PET scan characteristics of all scans classified as progressive metabolic disease (PMD) according to PERCIST. The table presents a comparison between the study-specific PMD subgroups: heterogeneous metabolic response (presence of both metabolic decrease > 30% (SUV) in ≥ 1 lesion and metabolic increase > 30% in ≥ 1 other lesion and/or appearance of new hypermetabolic lesions) and uniform progressive metabolic disease (followed standard PERCIST criteria without evidence of heterogenous metabolic response patterns)

	Heterogenous metabolic response, scans = 40	Uniform progressive metabolic disease, scans = 27	Progressive metabolic disease (PERCIST), scans = 67	*p* value
mBC subtype, n (column %)				
-Luminal A/B	32 (80.0%)	18 (66.7%)	50 (74.6%)	
-HER2-positive	1 (2.5%)	5 (18.5%)	6 (9.0%)	
-Triple negative	7 (17.5%)	4 (14.8%)	11 (16.4%)	0.08^a^
Histopathological subtype mBC, n (column %)				
-No specific type	30 (75.0%)	26 (96.3%)	56 (83.6%)	
-Lobular	7 (17.5%)	1 (3.7%)	8 (11.9%)	0.12^b^
-Other	2 (5.0%)		2 (3.0%)	
-Missing	1 (2.5%)		1 (1.5%)	
Lesion site at FDG-PET, n (column %)				
-Lymph node only	16 (40.0%)	12 (44.4%)	28 (41.8%)	
-Bone only	9 (22.5%)	1 (3.7%)	10 (14.9%)	
-Lymph nodes and bone	6 (15.0%)	3 (11.1%)	9 (13.4%)	
-Other combinations	9 (22.5%)	11 (40.7%)	20 (29.9%)	0.12^a^
Number of lesions at FDG-PET, n (column %)				
-1	0*	0	0	
-2	5 (12.5%)	5 (18.5%)	10 (14.9%)	
-3–5	14 (35.0%)	3 (11.1%)	17 (25.4%)	
-6–10	8 (20.0%)	8 (29.6%)	16 (23.9%)	
->10	13 (32.5%)	11 (40.7%)	24 (35.9%)	0.18^a^
Oligometastatic at FDG-PET **, n (%)				
-yes	16 (40.0%)	7 (25.9%)	23 (34.3%)	
-no	24 (60.0%)	20 (74.1%)	44 (65.7%)	0.30^b^
Current systemic treatment				
-Chemotherapy ± other therapy	18 (45.0%)	11 (40.7%)	29 (43.2%)	
-Endocrine therapy ± other therapy	13 (32.5%)	10 (37.0%)	23 (34.3%)	
-HER2 targeted therapy only	2 (5.0%)	1 (3.7%)	3 (4.5%)	
-PARP inhibitor only	5 (12.5%)	1 (3.7%)	6 (8.9%)	
-Antibody-Drug Conjugate only	0 (0.0%)	2 (7.4%)	2 (3.0%)	
-Other	2 (5.0%)	2 (7.4%)	4 (6.0%)	0.45^a^

*Per definition

**Definition: Up to five lesions, involving no more than three organs, and lesion size ≤ 5 cm

^a^ Chi‑square test assessing differences between the heterogeneous metabolic response subgroup and the uniform progressive metabolic disease subgroup

^b^ Fisher’s exact test assessing differences between the heterogeneous metabolic response subgroup and the uniform progressive metabolic disease subgroup

Abbreviations: mBC, metastatic breast cancer; HER2, human epidermal growth factor 2 receptor

Among CT-examinations, 65 of 141 (46%) follow-up examinations (Fig. 1, red box) had RECIST 1.1-measurable disease (one or more target lesion). Of the 38 examinations classified as progressive disease according to RECIST 1.1, 11 (29%) contained measurable target lesions and were therefore eligible for assessment of mixed response. None of those CT examinations fulfilled the mixed response criteria (0/11, 0%; Table 2).

The lesion counts on FDG‑PET were similar between scans with heterogeneous metabolic response (1, 2, 3–5, 6–10, and >10 lesions in 0, 5, 14, 8, and 13 scans, respectively) and those with uniform progressive metabolic disease (0, 5, 3, 8, and 11 scans in the same lesion categories; *p* = 0.18) (Table 3). The prevalence of oligometastatic disease was likewise comparable between groups (16 vs. 7 scans; *p* = 0.35). Thus, heterogeneous metabolic response was not associated with a more disseminated pattern of progression in this cohort.

## Discussion

In this retrospective real-world cohort study, we evaluated heterogeneous treatment response in mBC using paired FDG-PET and CT scans. FDG-PET identified heterogeneous response in a substantial proportion of examinations; notably, 60% of scans classified as PMD by PERCIST were reclassified as heterogeneous response based on lesion-level progression and regression. In contrast, CT detected no mixed responses using RECIST 1.1-derived criteria. These findings highlight the greater sensitivity of metabolic imaging for detecting lesion-level response variation during systemic therapy in mBC. The ability of FDG-PET to reveal heterogenous response patterns may provide a more nuanced reflection of tumor biology and treatment dynamics, supporting its potential value in response assessment for patients with mBC.

In addition to characterizing overall response categories, the present analysis also examined lesion‑level features and the characteristics of the subgroup exhibiting heterogeneous response. Specifically, a slightly higher proportion of luminal mBC was observed in the heterogeneous metabolic response subgroup, whereas histopathological subtype, lesion distribution, lesion counts, and the prevalence of oligometastatic disease were comparable across groups. These complementary analyses, which to our knowledge have not been conducted previously in this context, provide further insight into the biological underpinnings of heterogeneous metabolic behavior and highlight that such discordant response patterns often occur in the context of multifocal metastatic spread and across multiple metastatic sites.

### Previous studies

To the best of our knowledge, this study represents the largest cohort to date evaluating heterogeneous and mixed treatment response patterns using FDG‑PET and CT in mBC. In a previous study of 25 patients with bone dominant mBC, heterogeneous metabolic response was reported in 48% of FDG-PET examinations [19], compared with 30% of all FDG-PET response assessments in the present cohort of 51 patients. A prior study of 31 patients reported heterogeneous treatment response on FDG-PET but stable disease on CT [25]. Despite differing definitions of heterogeneity, most patients in our cohort were classified as progressive disease by RECIST, with only a minority as stable disease. Collectively, these findings underscore both the relevance and the frequency of this response phenotype and further support the clinical importance of recognizing lesion level variability when evaluating treatment efficacy in systemic therapy for mBC.

### Are we ready to consider metabolic response heterogeneity in mBC?

As systemic therapies become more diverse and biologically targeted, response assessment, particularly imaging, must move beyond binary classifications of response or progression. Instead, treatment evaluation requires a personalized and adaptive approach that balances therapeutic efficacy, toxicity, quality of life, and preservation of future options. Given the substantial proportion of scans classified as heterogenous metabolic response, the current PERCIST categorization may insufficiently reflect biologically relevant response patterns.

The concept of metastatic drift, driven by prolonged survival and sequential therapy, provides a plausible explanation for these findings. Over time, selective pressure promotes clonal diversification, such that some lesions remain treatment-sensitive while others acquire resistance, particularly in hormone receptor–positive mBC with a prolonged disease course [26]. The coexistence of both responsive and resistant lesions may represent a biologically distinct state with a more favorable prognosis than widespread, uniform progression [17–19], potentially reflecting partial treatment efficacy that still exerts meaningful disease control.

Recognizing this pattern underscores the need to consider whether heterogeneous response should be more explicitly addressed within future response frameworks for both clinical trials and clinical decision-making. However, prospective validation in larger cohorts is required to determine its full prognostic and therapeutic implications.

### Other potential response assessment methods

The role of whole-body diffusion-weighted magnetic resonance imaging in characterizing heterogeneous response warrants investigation. This modality is recognized for its high sensitivity in visualizing metastatic lesions, particularly in bone [27] and may offer complementary value in response assessment [28]; however, its use in this setting remains insufficiently studied. Beyond imaging, circulating tumor DNA (ctDNA) can provide a systemic, dynamic measure of clonal evolution that potentially complements lesion-level metabolic and anatomical imaging [29].

### Methodological considerations

This study has several limitations. Its retrospective design, limited sample size (although twice as large as previous study cohort), and non-randomized patient selection introduce a risk of selection bias, enriching the cohort for FDG-avid disease and cases in which CT assessment is challenging. Consequently, patients with FDG-avid disease and cases in which FDG-PET was considered clinically informative are likely overrepresented, whereas patients with low-FDG-avid disease may be underrepresented. Furthermore, because FDG-PET was primarily performed in clinically selected patients with inconclusive or discordant findings on conventional imaging, this may have influenced the observed distribution of RECIST 1.1 and PERCIST response categories, including the relatively high proportion of PMD examinations. In addition, standardized criteria for defining heterogeneous response on FDG-PET and mixed response on CT are not yet established; in this study, previously published definitions were applied to ensure methodological consistency. However, as heterogeneous metabolic response is not an established PERCIST category, alternative definitions, including an upper limit on the number of new lesions, may have yielded different prevalence estimates. The lack of CT examinations classified as mixed response could also be due to the mixed response criteria being based on RECIST 1.1, which limits the possibility of evaluating common manifestations such as small lymph nodes, bone-only, and bone-predominant disease. More refined mixed response criteria, where for example non-target lesions are considered, could potentially better capture the heterogeneity on CT but need to be defined and evaluated. Interpretation bias cannot be excluded, as both the original clinical CT readers and the study-specific reviewers had access to corresponding FDG-PET images and nuclear medicine reports, which may have influenced lesion detection. The baseline examination was defined according to the study inclusion and not according to treatment initiation. As a result, the study population included patients at different stages of systemic therapy, which may limit direct comparison with cohorts evaluated from the start of a treatment line. Furthermore, histopathological confirmation was not available for all imaging-detected lesions. Response assessments were initially performed by a final-year medical student under supervision, with full access to all imaging data and original clinical reports. While all FDG-PET categorizations and a predefined random subset of CT assessments were reviewed by two experienced readers as part of a predefined data monitoring plan, the study did not include independent duplicate review of all examinations. Consequently, interobserver variability was not formally assessed.

## Conclusion

In this real-world cohort of mBC, FDG-PET identified heterogeneous metabolic treatment response in a substantial proportion of patients; however, under current PERCIST criteria, these cases were uniformly classified as progressive metabolic disease. In contrast, using RECIST-inspired criteria, CT did not classify any patient as mixed response, largely due to the high prevalence of non-measurable, non-target lesion disease limiting RECIST-based assessment. These findings suggest that existing response frameworks may insufficiently reflect intra-patient heterogeneity in routine practice, a possibility that may be particularly relevant in luminal mBC.

While RECIST 1.1 and PERCIST remain essential for standardization, refinement of response strategies, particularly those integrating metabolic imaging, may be warranted to better capture biologically relevant response patterns and support individualized treatment decisions. As heterogeneous response is not an established PERCIST response category, its definition and potential role in future response assessment frameworks require further study.

## Electronic supplementary material

Below is the link to the electronic supplementary material.

Supplementary Material 1

## Data Availability

The data are not publicly available due to restrictions by Swedish and European law to protect patient privacy.

## Abbreviations

CT: Computed tomography
CMR: Complete metabolic response
CR: Complete response
FDG-PET: ^18^F fluorodeoxyglucose positron emission tomography
mBC: Metastatic breast cancer
PD: Progressive disease
PERCIST: PET Response Criteria in Solid Tumours
PMD: Progressive metabolic disease
PMR: Partial metabolic response
PR: Partial response
RECIST: Response Evaluation Criteria in Solid Tumours
SD: Stable disease
SMD: Stable metabolic disease
SUL: Standardized uptake value corrected for lean body mass
SUV: Standardized uptake value

## Declarations

### Critical relevance statement

FDG-PET revealed heterogeneous metabolic response in 60% of scans classified as progressive metabolic disease by PERCIST in metastatic breast cancer, whereas paired CT using RECIST‑derived criteria detected no mixed responses, highlighting limitations of current response frameworks.
