# Supplementary material for: Heterogeneous treatment response in metastatic breast cancer: discordance between FDG-PET and CT classification
Source: Cancer Imaging. 2026 Aug 26;26:114. doi: 10.1186/s40644-026-01119-4 (PMC13520370; doi:10.1186/s40644-026-01119-4)
Supplement: Supplementary file 1 [file 40644_2026_1119_MOESM1_ESM.docx]

Additional File 1. Comparison of heterogeneous metabolic response and uniform progressive metabolic disease - Detailed description of lesion sites

|  | Heterogenous metabolic response, scans = 40 | Uniform progressive metabolic disease, scans = 27 | Progressive metabolic disease (PERCIST), scans = 67 |
| --- | --- | --- | --- |
| Lesion site, n (column %) |  |  |  |
| - Lymph nodes only | 16 (40.0%) | 12 (44.4%) | 28 (41.8%) |
| - Bone only | 9 (22.5%) | 1 (3.7%) | 10 (14.9%) |
| - Lymph nodes and bone | 6 (15.0%) | 3 (11.1%) | 9 (13.4%) |
| - Lymph nodes and lung | 3 (7.5%) | 1 (3.7%) | 4 (6.0%) |
| - Lymph nodes, bone, and lung | 3 (7.5%) | 1 (3.7%) | 4 (6.0%) |
| - Lymph nodes, bone, and liver | 2 (5.0%) | 1 (3.7%) | 3 (4.5%) |
| - Bone and liver | 1 (2.5%) | - | 1 (1.5%) |
| - Bone, lymph nodes, liver, and pleura | - | 1 (3.7%) | 1 (1.5%) |
| - Bone, lymph nodes, liver, and lung | - | 3 (11.1%) | 3 (4.5%) |
| - Lymph nodes, bone, and muscle | - | 1 (3.7%) | 1 (1.5%) |
| - Liver only | - | 1 (3.7%) | 1 (1.5%) |
| - Lymph nodes and breast | - | 1 (3.7%) | 1 (1.5%) |
| - Lymph nodes, breast, and thoracic wall | - | 1 (3.7%) | 1 (1.5%) |
